# Supplementary material for: A Brownian ratchet model for DNA loop extrusion by the cohesin complex
Source: eLife. 2021 Jul 26;10:e67530. doi: 10.7554/eLife.67530 (PMC8313234; doi:10.7554/eLife.67530)
Supplement: Supplementary file 1. — The values and units of the parameters are indicated, together with an indication of changes between the simulated gripping and slipping states. [file elife-67530-supp1.docx]

**Supplementary File 1.** Cohesin-DNA interaction parameters in gripping and slipping states.

| Name | Description | Value (Gripping) | Value (Slipping) |
| --- | --- | --- | --- |
| *L*_p_ | DNA persistence length | 50 nm | no change |
| *D* | DNA segment length | 5 nm | no change |
| *N* | Number of DNA segments | 45 - 70 | no change |
| *L_A_* | Smc1/3 head-to-elbow distance | 30 nm | no change |
| *U_A_* | Smc1/3 elbow-to-hinge distance | 20 nm | no change |
| *H_A_* | Equilibrium Smc head-to-head distance | 4 nm | 14 nm |
| $\xi$ (See Eq. 4) | Head-to-head additional stiffness factor | 5 – 50 | no change |
| *K*_hinge_ | Parameter that characterizes stiffness of head-to-hinge interaction | 0.5 pN/nm | no change |
| $\theta$ | Equilibrium angle between head-to-elbow and elbow-to-hinge cohesin segments | 0 or 180 | 0 |
| $\alpha$ (See Eq. 5) | Stiffness of the slipping interaction between DNA and Smc3 | 5 pN/nm | no change |
| $\gamma$ (See Eq. 5) | Stiffness of the gripping interaction between DNA and Smc3 | 5 pN/nm | 0 |
| $\delta$ (See Eq. 5) | Stiffness of the interaction between DNA and the hinge | 5 pN/nm | no change |
